# *Bartonella* infections in cats and dogs including zoonotic aspects

**DOI:** 10.1186/s13071-018-3152-6

**Published:** 2018-12-04

**Authors:** Alejandra Álvarez-Fernández, Edward B. Breitschwerdt, Laia Solano-Gallego

**Affiliations:** 1 Departament de Medicina i Cirurgia Animals, Facultat de Veterinària, Universitat Autònoma de Barcelona, 08193 Bellaterra, Spain; 2 Department of Clinical Sciences and the Comparative Medicine Institute, College of Veterinary Medicine, North Carolina State University, 1060 William Moore Dr, Raleigh, NC 27607 USA

**Keywords:** *Bartonella*, Dog, Cat, Europe, Zoonosis

## Abstract

Bartonellosis is a vector-borne zoonotic disease with worldwide distribution that can infect humans and a large number of mammals including small companion animals (cats and dogs). In recent years, an increasing number of studies from around the world have reported *Bartonella* infections, although publications have predominantly focused on the North American perspective. Currently, clinico-pathological data from Europe are more limited, suggesting that bartonellosis may be an infrequent or underdiagnosed infectious disease in cats and dogs. Research is needed to confirm or exclude *Bartonella* infection as a cause of a spectrum of feline and canine diseases. *Bartonella* spp. can cause acute or chronic infections in cats, dogs and humans. On a comparative medical basis, different clinical manifestations, such as periods of intermittent fever, granulomatous inflammation involving the heart, liver, lymph nodes and other tissues, endocarditis, bacillary angiomatosis, peliosis hepatis, uveitis and vasoproliferative tumors have been reported in cats, dogs and humans. The purpose of this review is to provide an update and European perspective on *Bartonella* infections in cats and dogs, including clinical, diagnostic, epidemiological, pathological, treatment and zoonotic aspects.

## Background

*Bartonella* is a genus of Alphaproteobacteria within the family *Bartonellaceae*. *Bartonella* spp. are small, thin, short and slightly curved, gram-negative, hemotropic and rod-shaped bacteria [1]. They are catalase, oxidase, urease and nitrate reductase negative [1]. *Bartonella* spp. are fastidious, slow growing and facultative intracellular pathogens that are highly adapted to a broad spectrum of mammalian reservoir hosts and are mainly transmitted by arthropod vectors [2, 3]. Thirty-eight different *Bartonella* species have been isolated or detected from humans or from domestic and wild animals including bats, birds, canids, cattle, deer, felids, horses, marine mammals, rodents, sheep and reptiles [4–10]. *Bartonella* spp. are distributed throughout the world. In recent years, an increasing number of studies from around the world have reported canine and feline *Bartonella* infections. The purpose of this review is to provide an update while emphasizing European literature relative to *Bartonella* spp. infections in cats and dogs, including clinical, diagnostic, epidemiological, pathological, treatment and zoonotic aspects.

## Clinically relevant *Bartonella* species described in cats, dogs and humans

At least thirteen *Bartonella* species or subspecies have been recognized as agents of human disease, three species are reportedly responsible for the majority of clinical illness: *B*. *bacilliformis*, *B. quintana* and *B. henselae* [11]. Because serological testing for other *Bartonella* spp. is rarely performed in human medicine and due to difficulties associated with isolation or PCR amplification of these bacteria from patient specimens, it is possible that *B. koehlerae* [12, 13], *B. vinsonii berkhoffii*, as well as other species are under-recognized as a cause of human illness [14, 15].

Primary reservoirs, accidental hosts and the confirmed or suspected vectors for the main *Bartonella* species infecting cats and dogs with zoonotic potential are listed in Table 1. The most relevant species implicated in companion animal medicine are *B. clarridgeiae*, *B. elizabethae*, *B. henselae*, *B. koehlerae*, *B. quintana*, *B. rochalimae* and *B. vinsonii berkhoffii*. All of these species have been associated with severe illnesses in cats or dogs and all have zoonotic potential [16, 17]. Each *Bartonella* spp. appears to have co-evolved with a specific primary reservoir host which poses a source of infection for accidental hosts under natural exposure conditions [16].

**Table 1:** *Bartonella* specie*s* that infect cats and dogs with zoonotic potential including primary reservoir, accidental host and vectors

*Bartonella* species	Primary reservoir	Vector	Accidental host
*B. bovis* (ex *weissii*)	Domestic cattle (*Bos taurus*)	Biting flies, ticks	Humans, cats, dogs
*B. volans-*like	Flying squirrel (Pteromyinae)	Fleas	Humans, dogs, horses
*B. clarridgeiae*	Cats (*Felis catus*)	Cat flea (*Ctenocephalides felis*), ticks^a^	Humans, dogs
*B. elizabethae*	Rats (*Rattus norvegicus*)	Fleas	Humans, dogs
*B. grahamii*	Rodents	Rodent flea (*Ctenophthalmus nobilis*)	Humans, dogs
*B. henselae*	Cats (*Felis catus*), dogs (*Canis familiaris*)	Fleas, ticks^a^	Human, dogs
*B. koehlerae*	Cats (*Felis catus*), gerbils (*Meriones lybicus*)	Fleas	Humans, dogs
*B. quintana*	Humans, gerbils (*Meriones lybicus*)	Human body lice, fleas, bed bugs, pigeon mites^a^	Cats, dogs, monkeys
*B. rochalimae*	Canids	Fleas^a^ (*Pulex irritans*, *Pulex simulans*), ticks^a^	Humans, dogs
*B. vinsonii arupensis*	Rodents	Deer tick (*Ixodes scapularis*)	Humans, dogs
*B. vinsonii berkhoffii*	Coyotes (*Canis latrans*), dogs (*Canis familiaris*), foxes (*Urocyon* spp.)	Ticks^a^, *Pulex* spp.^a^	Humans
*B. washoensis*	California ground squirrel (*Spermophilus beecheyi*), rabbits (*Oryctolagus cuniculus*)	Fleas, ticks^a^	Humans, dogs

^a^Not confirmed

Details included in the table are provided in [16, 25, 40, 53, 57, 132, 175–183]

The cat (*Felis catus*) is the primary but not sole reservoir for *B. henselae* [18], the causal agent of human cat scratch disease (CSD). Domestic cats are also principal reservoir hosts for *B. clarridgeiae* and *B. koehlerae.* Infected cats are thought to rarely develop clinical signs [19]. However, chronic, relapsing bacteremia can frequently be detected in infected cats and potential long-term consequences of relapsing bacteremia are unknown [20–22]. Cats can also be infected with *B. bovis* (ex *weissii*) and *B. quintana*, but the role of domestic cats in the epidemiology of these two *Bartonella* species has not been clearly established [21].

The dog (*Canis familiaris*) may also be a host for *B. henselae* and canines are considered the primary reservoirs for *B. vinsonii berkhoffii*, causing endocarditis in dogs and humans [23, 24]. Wild canids such as coyotes (*Canis latrans*) in California and potentially domestic dogs have been described as main reservoir hosts for *B. vinsonii berkhoffii*, as prolonged bacteremia also occurs in these animals [5, 25, 26]. *Bartonella henselae*, first isolated from a dog in Gabon in 2003 may be the *Bartonella* spp. that most often infects pet dogs [27]. Dogs can also be infected with *B. clarridgeiae*, *B. elizabethae*, *B. koehlerae*, *B. quintana*, *B. rochalimae* and *B. washoensis*, potentially causing similar disease manifestations as reported in humans, including bacillary angiomatosis, endocarditis, granulomatous hepatitis and granulomatous lymphadenitis, myocarditis, peliosis hepatis and others [20, 28–32]. Due to direct and frequent contact with humans, pet and stray infected cats and dogs pose a potential risk for human infection [33].

*Bartonella henselae* also causes multiple other clinical entities in human patients, potentially related to the individual’s immune status, variations in strain virulence, co-infection with other pathogens and co-morbidities [34]. Infection with *B. clarridgeiae* has been suspected in a few CSD cases and the organism has been isolated from one asymptomatic human blood donor [35]. *Bartonella koehlerae* has been associated with regional pain syndrome type I [13], hallucinations, sensory neuropathy, peripheral visual deficits [36], endocarditis [22] and other clinical conditions [12]. *Bartonella vinsonii berkhoffii* has been associated with human endocarditis and a spectrum of neurological symptoms [23, 24]. *Bartonella quintana*, the agent of trench fever, has been classically considered a human-specific species transmitted solely by human body lice [37]. However, *B. quintana* DNA has been detected in dogs with endocarditis [38] and healthy dogs [39, 40], cats [41, 42] and monkeys (*Macaca fascicularis* and *Macaca mulatta*) [43, 44].

## Inter- and intra-species transmission

Intra-erythrocytic *Bartonella* organisms within the bloodstream are ingested by blood-sucking arthropod vectors, mainly fleas, lice, sand flies, biting flies and ticks, after which they are transmitted to a primary reservoir or to an accidental host [37] (Table 1). Vector transmission occurs in two primary ways: (i) inoculation of *Bartonella-*contaminated arthropod feces *via* animal scratches or bites or by self-inflected contamination of wounds induced by the host scratching irritating arthropod bites. These are important modes of transmission among primary reservoir and accidental hosts, including cats, dogs and humans [45–47]. (ii) The other primary mode of transmission is by vector bites, as confirmed for *Lutzomyia verrucarum* sand flies, the vector of *B. bacilliformis* among humans [48]. Experimentally, using an *in vitro* model, *Ixodes ricinus* ticks were able to infect mammalian blood with *B. henselae* [49]. Furthermore, the presence of *Bartonella* spp. DNA, particularly *B. henselae*, has been well documented in questing ticks from Europe and other continents [50–52]. Ticks have also been clinically implicated in the transmission of *Bartonella* infection to dogs or humans in the absence of other vectors or known modes of transmission [53–56]. Interestingly, regurgitation of *B. henselae* by cat fleas (*Ctenocephalides felis*) has been demonstrated experimentally [57], but additional studies are needed to confirm flea-bite transmission to animals or humans. It is important to note that non-vectorial modes of transmission are also possible such as transmission through needle stick injury to veterinarians [58] or by blood transfusion as documented experimentally in cats, dogs and humans [59–62].

## Epidemiology, prevalence and distribution in Europe

**Fig. 1:**
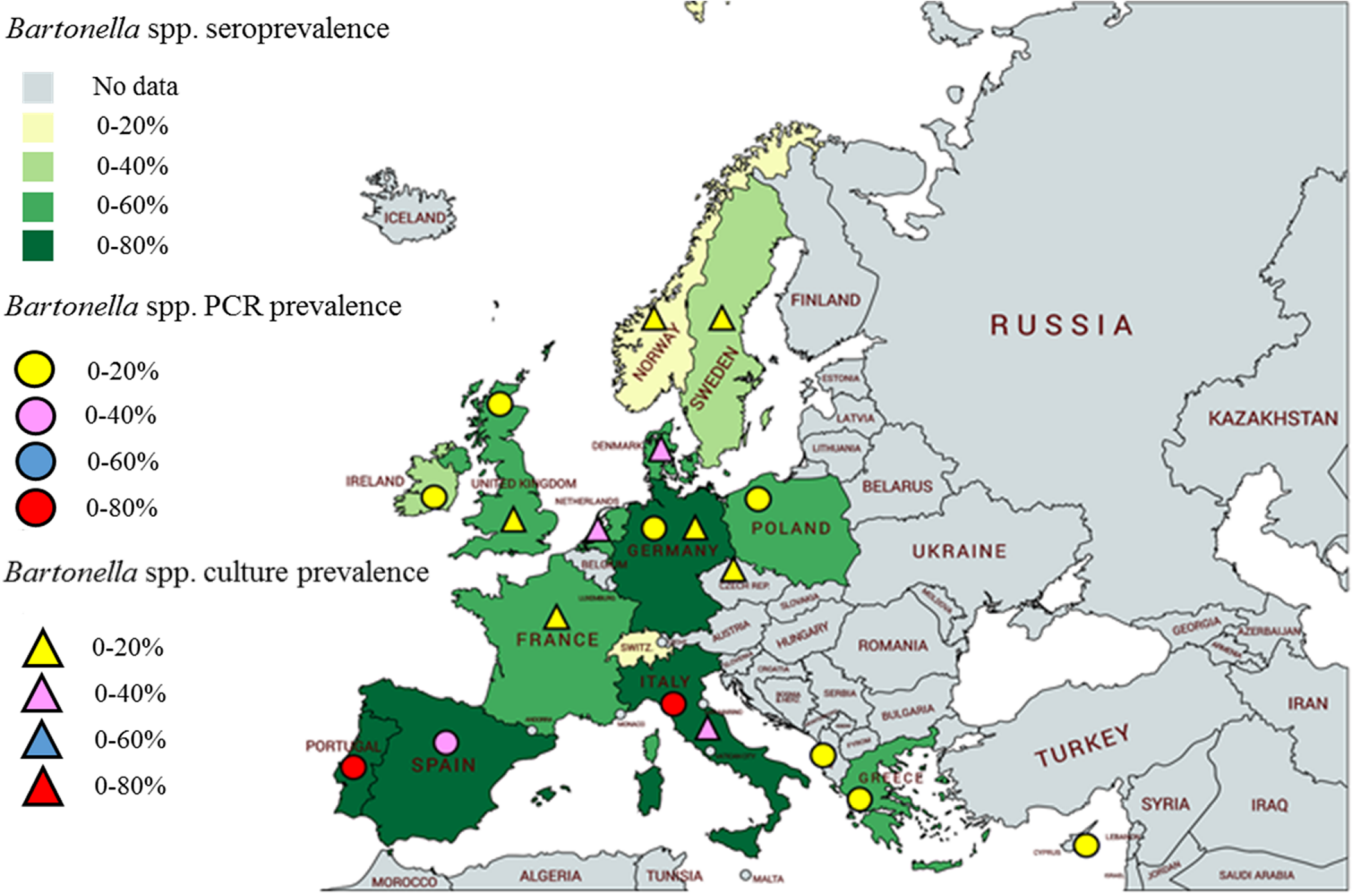
Geographical distribution maps depicting *Bartonella* serological, molecular and culture prevalences in cats from European countries. Information provided based on clinico-epidemiological studies reported in Table 2. Created with mapchart.net

**Fig. 2:**
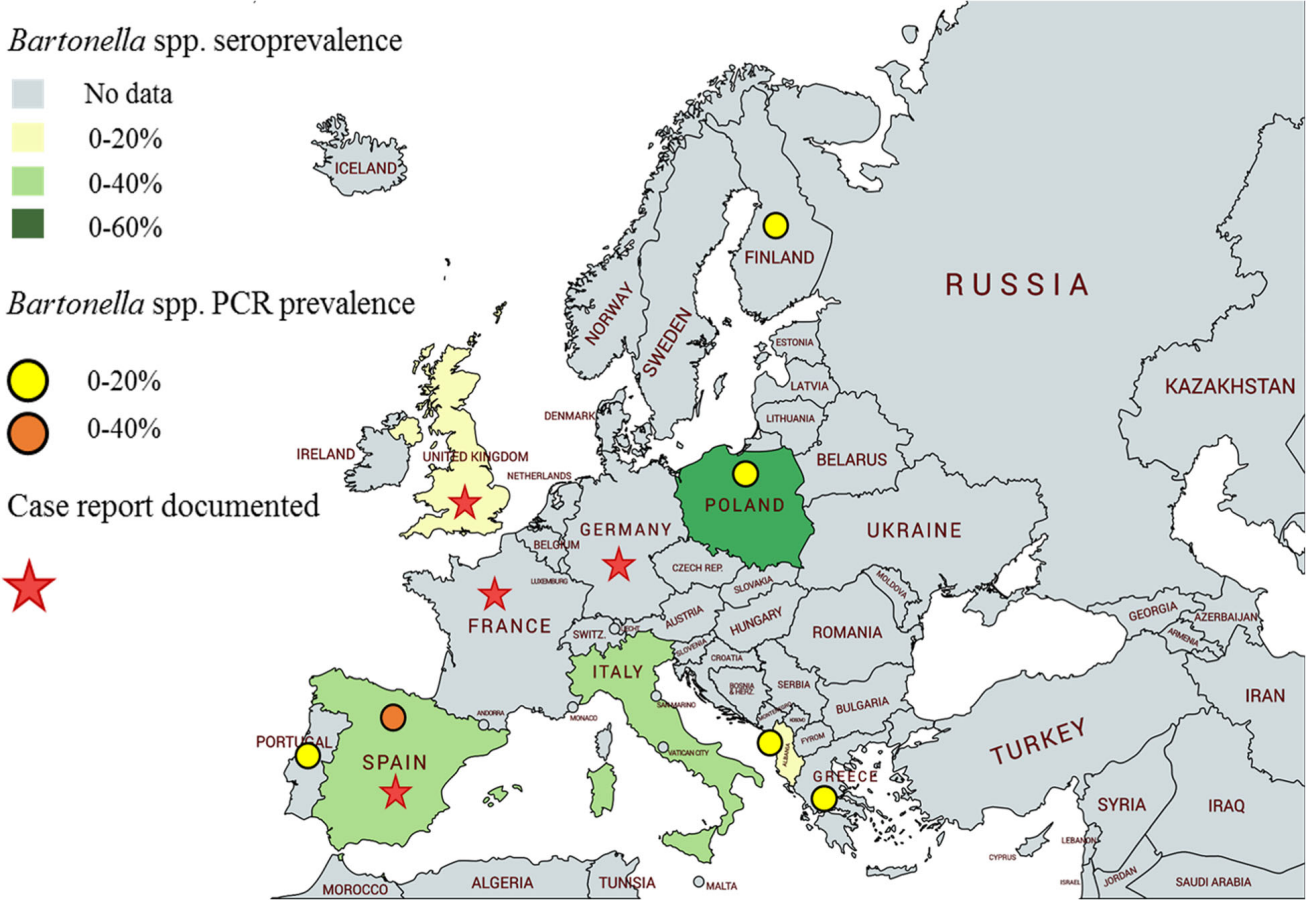
Geographical distribution maps depicting *Bartonella* serological and molecular prevalences in dogs from European countries as well as documented case reports. Information provided based on clinico-epidemiological studies reported in Table 3. Created with mapchart.net

Serology, PCR or culture-based clinico-epidemiological studies in cats and dogs in Europe are summarized in Tables 2 and 3 and Figs. 1 and 2. More than 50 feline and canine seroprevalence studies have been reported from different European countries (Tables 2, 3); however, culture or PCR confirmed cases of canine or feline bartonellosis have been infrequently reported. *Bartonella* spp. seroprevalence rates are high in cats in European Mediterranean countries, where temperature and humidity are favorable for flea and tick infestations [20] (Fig. 1). In Europe, *Bartonella* antibody prevalence in cats ranges from 0% in Norway [63] to 71.4% in Spain [64] (Table 2). Bacteremic prevalence rates for various combinations of *B. clarridgeiae*, *B. henselae* and *B. koehlerae* often approach 50–75% in feral cat populations worldwide [17]. Generally, the differences in serological or bacteremic prevalences are related to different climatic conditions, whether the cat population tested consisted of pet or stray cats and whether acaricide products were used routinely. Information regarding clinic-epidemiological studies performed in cats in other continents is summarized in Table 4.

**Table 2:** *Bartonella* spp. clinico-epidemiological studies involving cats in Europe

Country (area, year)	Total no. of animals studied (lifestyle)	Percentage of positive animals			Confirmed *Bartonella* spp. and type using molecular methods	Reference
		Serology (method or antigen used)^a^	Blood PCR	Blood culture		
Albania (Tirana, 2014)	146 (client-owned)	nr	0.7	nr	*B. henselae*	[182]
Cyprus (2017)	174 (stray and client-owned)	nr	10.9	nr	*B. henselae*	[183]
Greece (Crete, Mykonos, Skopelos, Athens, 2017)	148 (stray)	58.8	4.7	nr	*B. henselae*, *B. clarridgeiae*	[184]
Greece (Thessaly, Macedonia, 2018)	100 (client-owned)	nr	8.5	nr	*B. henselae*, *B. clarridgeiae*, *B. koehlerae*	[178]
Czech Republic (Prague, 2003)	61 (stray, client-owned and shelter)	nr	nr	8.0	*B. henselae* type II	[185]
Denmark (2002)	93 (stray and client-owned)	45.6	nr	22.6	*B. henselae* types I and II	[186]
France (Nancy, 1997)	94 (stray)	nr	nr	53	*B. henselae* types I and II, *B. clarridgeiae*	[187]
France (Paris, 2001)	436 (client-owned)	41.1	nr	16.5	*B. henselae* types I and II, *B. clarridgeiae*	[188]
France (Lyon, 2004)	99 (client-owned)	nr	nr	8.1	*B. henselae*, *B. clarridgeiae*	[189]
Germany (Freiburg, 1997)	100 (client-owned)	nr	nr	13	*B. henselae*	[190]
Germany (southern and northern, 1999)	713 (stray and client-owned)	1	nr	nr	nr	[191]
Germany (Berlin, 2001)	193 (client-owned and stray)	nr	nr	20	*B. henselae* types I and II, *B. clarridgeiae*	[192]
Germany (Hannover and others, 2011)	507 (nr)	68.7 (ELISA)	nr	2.2	*B. henselae*	[193]
Germany (north-east, 2012)	256 (stray and client-owned)	37.1; 18.8 (*B. quintana*)	0	nr	na	[194]
Germany (southern, 2017)	479 (nr)	nr	2.5	nr	*B. henselae*, *B. clarridgeiae*	[195]
Italy (Tuscany, 2002)	427 (client-owned and shelter)	16.0	4.0	0	*B. henselae*	[196]
Italy (northern, 2002)	248 (nr)	nr	nr	9.7	*B. henselae*	[197]
Italy (northern, 2004)	1585 (stray)	39.0	nr	2.0	*B. henselae* types I and II, *B. clarridgeiae*	[198]
Italy (Sassari, 2007)	79 (stray and client-owned)	21.5	nr	nr	na	[199]
Italy (Sardinia, 2009)	55 (nr)	10.9	5.5	nr	*B. henselae*	[73]
Italy (southern, 2010)	85 (client-owned)	nr	83.5	nr	*B. henselae*	[148]
Italy (Sicily, 2012)	182 (stray and client-owned)	57.1	nr	nr	na	[200]
Italy (Pisa, 2012)	234 (client-owned)	33.3	11.1	nr	*B. henselae* types I and II	[201]
Italy (northern, 2013)	1340 (stray)	23.1	nr	17.0	*B. henselae*, *B. clarridgeiae*	[202]
Italy (northern, 2016)	82 (stray)	30.4	nr	nr	na	[203]
Italy (southern, 2016)	42 (nr)	54.8	38.1	nr	*B. henselae*, *B. clarridgeiae*	[204]
Italy (Aeolian Islands, 2017)	330 (client-owned)	nr	3.9	nr	*B. henselae*, *B. clarridgeiae*	[205]
Ireland (Dublin area, 2010)	121 (client-owned)	26.5 (ELISA)	5.2	nr	*B. henselae* type II, *B. clarridgeiae*	[206]
Netherlands (1997)	163 (stray and client-owned)	51.8 (ELISA)	nr	22.0	*B. henselae*	[207]
Norway (2002)	100 (stray and client-owned)	0	nr	0	na	[63]
Poland (Varsaw, 2007)	137 (nr)	45.0	10.2	nr	*B. henselae*, *B. clarridgeiae*	[208]
Portugal (Lisbon, Evora, 2009)	51 (client-owned, shelter and stray)	64.9	67.7	nr	*B. henselae*	[209]
Portugal (1995)	14 (nr)	14.3 (*B. quintana*); 6.7	nr	nr	na	[210]
Portugal (2014)	649 (stray and client-owned)	nr	2.9	nr	*Bartonella* spp.	[211]
Spain (Barcelona, Tarragona, Mallorca, 2005)	115 (client-owned)	29.6	7.0	nr	*B. henselae*	[212]
Spain (Barcelona, Tarragona, Mallorca, 2006)	168 (client-owned)	71.4	17.0	nr	*B. henselae*, *B. clarridgeiae*	[64]
Spain (Barcelona, 2008)	100 (client-owned)	nr	1	nr	*B. clarridgeiae*	[213]
Spain (Madrid, 2012)	680 (client-owned and stray)	24.7	0.3	nr	*B. henselae*	[127]
Spain (Rioja, Catalonia, 2013)	147 (stray and client-owned)	nr	32	nr	*B. henselae*, *B. clarridgeiae*	[214]
Spain (multiple locations, 2015)	86 (client-owned)	50	nr	nr	*B. henselae*	[215]
Spain (Zaragoza, 2016)	47 (stray and shelter)	nr	38.29	nr	*B. henselae*	[216]
Spain (Catalonia, 2016)	116 (shelter)	35.3 (ELISA)	22.4	nr	*B. henselae*, *B. clarridgeiae*	[217]
Scotland (2011)	52 (client-owned and stray)	15.4 (ELISA)	5.8	nr	*B. henselae*	[218]
Sweden (different locations, 2002)	292 (nr)	0 (*B. quintana*); 25 (*B. elizabethae*); 1 (*B. henselae*)	nr	nr	na	[219]
Sweden (Stockholm, 2003)	91 (client-owned)	nr	nr	2.2	*B. henselae* type II	[220]
Switzerland (Tessin, northern, 1997)	728 (client-owned and shelter)	8.3	nr	nr	na	[150]
UK (Bristol, 2002)	360 (nr)	nr	nr	9.4	*B. henselae* types I and II	[221]
UK (2000)	148 (stray and client-owned)	41.2 (ELISA)	nr	nr	na	[72]

^a^Tested by IFA for *B. henselae* antigen unless another method or antigen is indicated

*Abbreviations*: *ELISA* enzyme-linked immunosorbent assay, *IFA* indirect immunofluorescence assay, *na* not applicable, *nr* not reported

**Table 3:** *Bartonella* spp. clinico-epidemiological studies performed in European dogs

Country (area, year)	Total no. of animals studied (lifestyle)	Percentage of positive animals^a^		Confirmed *Bartonella* spp. and type using molecular methods	Reference
		Serology (method or antigen used)^b^	Blood PCR		
Albania (Tirana, 2009)	30 (stray)	0 (ELISA)	0	na	[222]
Finland (southern, 2014)	390 (client-owned and hunting)	nr	0	na	[223]
Greece (Thessaloniki, 2009)	50 (client-owned sick)	nr	4	*B. rochalimae*, *Bartonella* strain HMD	[32]
Italy (Sassari, 2007)	58 (shelter, client-owned)	28.3	nr	na	[199]
Italy (Bologna, 2007)	381 (client-owned)	6	nr	na	[224]
Italy (Basilicata, Ginosa, 2009)	60 (shelter and client-owned)	6.6; 1.7 (*B. vinsonii berkhoffii*)	11.6	*B. henselae*, *B. vinsonii berkhoffii* types I and II, *Bartonella* strain HMD	[32]
Italy (Sardinia, 2009)	190 (nr)	9.5	0	na	[73]
Italy (Aeolian Islands, 2017)	263 (client-owned)	nr	0	na	[205]
Poland (Warsaw, 2007)	54 (nr)	5.0; 5.5 (*B. vinsonii berkhoffii*)	10.2	*B. henselae*, *B. vinsonii berkhoffii*	[208]
Poland (northwestern, 2011)	242 (client-owned and shelter)	nr	0.3	*Bartonella* spp.	[225]
Portugal (southern, 2014)	1010 (client-owned and stray)	nr	0	na	[211]
Spain (northern, 2006)	466 (client-owned)	16.8; 1.1 (*B. vinsonii berkhofii*)	nr	na	[74]
Spain (Barcelona, 2009)	153 (nr)	nr	0	na	[226]
Spain (north-west, 2018)	61 (client-owned *Leishmania* infected sick dogs);	40	nr	na	[227]
	16 (client-owned healthy)	21			
Spain (north-west, north-east, south-east, 2018)	30 (client-owned dogs with culture negative endocarditis)	nr	26.6^c^	*B. rochalimae*, *B. vinsonii berkhoffii*, *B. koehlerae*	[136]
Spain (north-east, 2018)	68 (client-owned dogs with pericardial effusion)	nr	0^d^	na	[228]
UK (2000)	100 (client-owned)	3 (ELISA)	nr	na	[72]
UK (Bristol, 2002)	211 (nr)	nr	nr	na	[221]

^a^Blood culture was not performed in any of the listed studies with the exception of one study performed in Bristol that did not isolate *Bartonella* in dogs studied [221]

^b^Tested by IFA for *B. henselae* antigen unless another method or antigen is indicated

^c^Samples were from cardiac valve tissue and blood

^d^Samples were from pericardial effusion and blood

*Abbreviations*: *ELISA* enzyme-linked immunosorbent assay, *IFA* indirect immunofluorescence assay, *na* not applicable, *nr* not reported

**Table 4:** Summary of main clinico-epidemiological studies carried out in cats in continents other than Europe

Continent	Area or country	*Bartonella* spp. seroprevalence (%)	PCR/Culture prevalence (%)	Confirmed *Bartonella* spp. and type	Reference
Africa	Eastern	11	nf	nf	[229]
	Northern	15.0–59.6	PCR: 0.9–23.5; Culture: 17.0	*B. clarridgeiae*, *B. henselae*	[230–233]
	Southern	21.0–24.0	PCR: 7.8	*B. clarridgeiae*, *B. henselae*	[234–236]
Asia	China	nf	PCR: 10.5–21.5; Culture: 5.8–18.6	*B. clarridgeiae*, *B. henselae*	[237]
	Japan	8.8	PCR: 4.6; Culture: 2.0–20.0	*B. clarridgeiae*, *B. henselae*	[238–240]
	Korea	nf	PCR: 41.8–44.1	*B. clarridgeiae*, *B. henselae*	[241]
	Middle East	1.2–39.5	PCR: 9.4; Culture: 4.3–9.4	*B. clarridgeiae*, *B. henselae* type I, *B. koehlerae*	[242–246]
	Philippines	62.6–68.0	Culture: 61.0	*B. clarridgeiae*, *B. henselae*	[247]
	Thailand	nf	Culture: 12.8–50.0	*B. clarridgeiae*, *B. henselae* types I and II	[248]
Australia	Eastern	37	PCR: 26.2	*B. clarridgeiae*, *B. henselae*	[249]
	South New Wales (Sydney)	nf	Culture and PCR: 35.0	*B. henselae*	[250]
	Western and Dirk Hartog and Christmas islands	nf	PCR: 0–5.2	*B. henselae*, *B. koehlerae*	[250, 251]
North America	Centre	0–45.0	nf	nf	[252]
	East	10.0–85.2	PCR: 0–62.5	*B. clarridgeiae*, *B. henselae* types I and II, *B. koehlerae*	[243, 252–257]
	West	0–26.2	PCR: 27.0–27.7; Culture: 32.8	*B. clarridgeiae*, *B. henselae* types I and II, *B. koehlerae*	[252, 258, 259]
South America	Argentina	nf	PCR: 17.0	*B. clarridgeiae*, *B. henselae*	[260]
	Brazil	15–68	PCR: 4.5–97.0; Culture: 45.5	*B. clarridgeiae*, *B. henselae*	[261–264]
	Chile	5.6–8.0	PCR: 18.1; Culture: 41	*B. clarridgeiae*, *B. henselae*, *B. koehlerae*	[265, 266]
	Galapagos islands	75.0	PCR: 59.0	*B. clarridgeiae*, *B. henselae*	[267]
	Guatemala	nf	PCR: 33.8; Culture 8.2	*B. clarridgeiae*, *B. henselae*	[268]

*Abbreviations*: *PCR* polymerase chain reaction, *nf* not found

*Bartonella henselae* infection is commonly encountered in cats and potentially dogs and humans worldwide [65] (Tables 2, 3, 4, 5). *Bartonella clarridgeiae*, *B. quintana*, *B. koehlerae* and *B. bovis* are less frequently isolated from domestic cats than *B. henselae*, potentially because these species are more difficult to isolate or are unevenly distributed worldwide (Tables 2, 4). In Europe, *B. clarridgeiae* serological and molecular prevalence rates vary from 17 to 36%, while *B. quintana* seroprevalence rates range from 0 to 18%, among a few reported studies (Table 2). Interestingly, *B. koehlerae* and *B. bovis* have not yet been documented to infect cats in Europe although *B. koehlerae* DNA has been amplified from cat fleas in France [66].

**Table 5:** Summary of main clinico-epidemiological studies carried out in dogs in continents other than Europe

Continent	Area or country	*Bartonella* spp. seroprevalence (%)	PCR / culture prevalence (%)	Confirmed *Bartonella* spp. and type	Reference
Africa	East	nf	PCR: 0	nf	[269]
	Central	nf	PCR: 2.3	*B. clarridgeiae*, *B. henselae*	[27]
	North	19.5–47.4	PCR: 0.85–37.1	*B. clarridgeiae*, *B. elizabethae*, *B. henselae*, *B. rochalimae*, *B. vinsonii berkhoffii*	[230, 270–272]
	South	14	PCR: 0–9.0; Culture: 0	*B. henselae*	[233, 273, 274]
	West	nf	PCR: 0	nf	[275]
Asia	Middle East	6.6–47.4	Culture and PCR: 9.2–37.1	“*Candidatus* Bartonella merieuxii”, *B. vinsonii berkhoffii* (type III in one study)	[276–278]
	South Korea	nf	PCR: 0–29.6	*B. clarridgeiae*, *B. henselae*	[241, 279, 280]
	Sri Lanka	5.1	PCR: 3.38	*Bartonella* strain HMD	[281]
	Thailand	38	PCR: 1.8; Culture and PCR: 0.3–31.3	*B. clarridgeiae*, *B. elizabethae*, *B. grahamii*, *B. quintana*, *B. taylorii*, *B. vinsonii arupensis*	[40, 282–284]
	Vietnam	0	PCR: 0	nf	[281]
Australia	New South Wales and Northern Territory	0	Culture and PCR: 0	nf	[285]
North America	Centre	0–20	nf	nf	[69, 286]
	East	0–49	PCR: 9.2; Culture: 52.5	*B. bovis*, *B. henselae*, *B. koehlerae, B. vinsonii berkhoffiii*, *B. volans*-like	[68, 69, 128, 286]
	West	0–29	PCR: 0–1.7; Culture: 2.2	*B. clarridgeiae*-like, *B. vinsonii berkhoffii*	[69, 286–288]
South America	Argentina	nf	PCR: 3	*B. clarridgeiae*	[260]
	Brazil	1.5–24.8	PCR and culture: 1	*B. henselae*, *B. vinsonii berkhoffii*	[262, 264, 281, 289]
	Chile	nf	PCR: 4.3	*B. henselae*, *B. vinsonii berkhoffii*	[290]
	Colombia	10.1	PCR: 0.77	*B. rochalimae*, *B. vinsonii berkhoffii*	[281]
	Galapagos Islands	nf	PCR: 13.6	*B. clarridgeiae*, *B. elizabethae*, *B. henselae*	[267]
	Peru	40–62	PCR: 10	*B. rochalimae*, *B. vinsonii berkhoffii* type III	[291]

*Abbreviations*: *PCR* polymerase chain reaction, *nf* not found

*Bartonella* exposure or infection prevalence studies involving cats have been widely reported from around the world (Tables 2, 4), whereas fewer serological or isolation studies are available regarding *Bartonella* exposure or infection in dogs (Fig. 2). In the USA, one study found a 3.6 % *B. vinsonii berkhoffii* seroprevalence in 1920 clinically ill dogs. The *B. vinsonii berkhoffii* seroprevalence increased to 36 and 52% if the dogs were co-exposed to *Ehrlichia canis* or *Babesia canis*, respectively [67]. Another study found *B. henselae* IgG antibodies in 10.1% of healthy dogs and in 27.2% of sick dogs, whereas *B. vinsonii berkhoffii* IgG antibodies were detected in only 1% of healthy dogs and 4.7% of sick dogs [68]. A recent *Bartonella* seroepidemiology study from North America found overall low *B. henselae*, *B. koehlerae* and *B. vinsonii berkhoffii* seroprevalences in dogs in which a vector-borne disease was suspected [69]. In California, 102 out of 3417 (2.99%) sick dogs were seroreactive for at least one species of *Bartonella* antigen. Of these, 36 (35.3%) had antibodies against *B. henselae* only, 34 (33.3%) had antibodies against *B. clarridgeiae* only, 2 (2.0%) had antibodies against *B. vinsonii berkhoffii* only and 30 (29.4%) had antibodies against a combination of these antigens [70]. Although the sensitivity of *Bartonella* spp. indirect immunofluorescence assay (IFA) most likely under estimates overall seroprevalence, IFA specificity appears to approach 100% [71]. *Bartonella* seroprevalence data is more limited in Europe and other continents in dogs when compared to North America (Tables 3, 5). In Europe, 3% were *B. henselae*-seropositive in the UK [72] and 5.8% in Italy [73]. In Spain, *B. henselae* and *B. vinsonii berkhoffii* seroprevalences were 16.8 and 1.1%, respectively [74]. Moreover, it is important to remark that after a search in PubMed we found around seven times more reports of *Bartonella* infection in dogs in the USA than in Europe [5, 14, 28–31, 38, 75–100]. Information regarding clinico-epidemiological studies performed in dogs in other continents is summarized in Table 5.

The annual number of human cases of CSD in the USA is estimated to be 12,000 outpatients and 500 inpatients [101]. Comparative data have not been reported for European countries. By IFA testing, *B. henselae* seroprevalence rates reported for humans in Europe range between 3 and 30% [51, 102, 103]. A recent study that used six *Bartonella* spp. or genotype antigens to test 89 Spanish veterinarians documented a high *Bartonella* spp. seroprevalence (73.0%), as well as a high molecular prevalence (7.9%) using *Bartonella* alpha proteobacteria growth medium (BAPGM)/PCR [104]. Interestingly, the lowest IFA seroreactivity was found against *B. quintana* antigen (11.2%) and the highest, against *B. vinsonii berkhoffii* genotype III antigen (56%). Infection with *B. henselae*, *B. vinsonii berkhoffii* genotypes I and III, and *B. quintana* DNA was documented [104]. In a previous study carried out in Spain using a commercial enzyme-linked immunosorbent assay (ELISA) kit *B. henselae* seroprevalence in cat owners, and blood donors was 28.9 and 5.9%, respectively [103]. These lower percentages most likely reflect differences in the antigens used, and exposure risks among the study populations. It is relevant to remark that veterinary personnel have a major exposure risk compared to the general population [104].

## Infection and pathogenesis

In animal models, mainly cat and mouse, after initial inoculation the infection cycle of *Bartonella* spp. is initiated by colonization of the still enigmatic primary niche where the bacteria reside, persist and are periodically seeded into the bloodstream to cause the typical relapsing *Bartonella* spp. bacteremia [105]. Endothelial cells, lymph nodes, liver, spleen, kidney, dermis and the bone marrow are some of the proposed niches where *Bartonella* spp. have been isolated from mammals [106–112]. *Bartonella henselae* has been shown to infect erythrocytes, endothelial cells, macrophages, microglial cells and even human CD 34^+^ progenitor cells [113–116]. In a recent study, *Bartonella tribocorum* subcutaneous inoculated in rats led to bloodstream invasion through the lymphatic circulation [110], a finding that may have clinical implications for diseases such as chylothorax.

*Bartonella tribocorum* was able to resist macrophage phagocytosis and to inhibit pyroptosis at an early stage of infection [110]. Endothelial cells are an important target cell type in probably all mammals, including humans incidentally infected by zoonotic species [117]. The tropism of *Bartonella* spp. for endothelial cells in conjunction with their proximity to the bloodstream suggest that endothelial cells are an important constituent of the primary niche [118]. In mammalian reservoir hosts, *Bartonella* spp. infection is characterized by chronic intraerythrocytic bacteremia whereas in accidental hosts the bacteria are less frequently documented in association with intraerythrocytic bacteremia, potentially due to a very low-level infection of erythrocytes [105, 119]. *Bartonella* spp. are able to colonize endothelial cells in both, accidental and reservoir hosts [120]. The endothelial or vascular niche provides the bacterium with a means of seeding the blood with organisms on a sporadic basis, potentially contributing to infection of CD34^+^ progenitor cells in the bone marrow, as well as circulating erythrocytes and monocytes [16].

In dogs, *B. vinsonii berkhoffii* can induce vascular endothelial growth factor (VEGF) endothelial cell proliferations, as reported for *B. bacilliformis*, *B. henselae* and *B. quintana* in human patients [121] leading to vascular tumor formation [118] and vasoproliferation, particularly in patients with human immunodeficiency virus (HIV) or therapeutic suppression of the immune system [121]. There is *in vitro* evidence that *B. vinsonii berkhoffii* genotypes I, II and III are capable of inducing activation of hypoxia inducible factor-1 and production of VEGF, thereby providing mechanistic evidence as to how these bacteria could contribute to the development of vasoproliferative lesions [121]. For this reason, infection with one or more *Bartonella* spp. may contribute to the pathogenesis of systemic reactive angioendotheliomatosis and hemangiopericytomas in animals [14, 122]. In human patients, activation of hypoxia inducible factor-1 and production of VEGF have been reported for *B. bacilliformis*, *B. henselae* and *B. quintana* [121] leading to vascular tumor formation [118] and vasoproliferation, particularly in patients with HIV or therapeutic suppression of the immune system [121]. Moreover, in humans, *Bartonella* spp. infections range from low to no morbidity (e.g. reactive, suppurative, or granulomatous lymphoid response in immunocompetent individuals), to occasional mortality (e.g. *B. quintana* infection), to substantial mortality in Peru due to the severe hemolytic anemia associated with *B. bacilliformis* [123].

## Clinical signs and laboratory abnormalities

The spectrum of disease manifestations associated with *Bartonella* spp. infections continues to expand, yet remains incompletely characterized in many domestic and wild animals [16]. Although most acute *Bartonella* infections are probably self-limiting, persistent infections appear to be associated with a wide variety of clinical signs and pathological abnormalities in cats, dogs, and humans. *Bartonella* infections manifest from subclinical bacteremia (e.g. healthy animal and human blood donors) to encephalitis, fever of unknown origin, lymphadenomegaly, endocarditis and myocarditis, ocular disease (neuroretinitis, uveitis), skin inflammation and many other less common disease manifestations [124]. Some factors that could influence the appearance of disease manifestations include virulence differences among *Bartonella* spp. and strains, mode of transmission, differences in the host immune response, concurrent infectious or non-infectious diseases, immunosuppression and malnutrition [16, 125].

In the context of comparative medicine, One Health and pet ownership, *B. henselae*, *B. koehlerae* and *B. vinsonii berkhoffii* are the three *Bartonella* spp. most frequently associated with pathology in cats, dogs and humans. As reservoir host for *B. henselae* and *B. koehlerae*, cats can be sub clinically infected for months and even years [54]. However, more virulent strains of these species, as well as other *Bartonella* spp. for which cats are accidental hosts, appear to result in enhanced pathogenicity (Table 6). Furthermore, immunosuppressive viral infections like feline leukemia virus (FeLV) may predispose to *B. henselae* infection or persistence in cats [126] (Table 6). Despite long-standing bloodstream infection in cats, complete blood count, serum biochemical profiles and urinalysis findings are frequently normal; however, laboratory abnormalities reported with some frequency in sick cats include anemia, eosinophilia, hyperproteinemia, hyperglobulinemia, neutropenia and thrombocytopenia [127]. In cats experimentally infected with *B. henselae* by blood transfusion, histopathological lesions revealed peripheral lymph node hyperplasia, splenic follicular hyperplasia, lymphocytic cholangitis/pericholangitis, lymphocytic hepatitis, lymphoplasmacytic myocarditis and interstitial lymphocytic nephritis [112]. These indicators of chronic inflammation support the need for long-term studies to determine if cats (or other animals) suffer biological consequences for long-standing infection with one or more *Bartonella* spp.

**Table 6:** Clinical sign, lesions and laboratory abnormalities reported in association with *Bartonella* infections in cats, dogs and humans

*Bartonella* spp*.*	Hosts^a^		
	Cats	Dogs^b^	Humans
*B. henselae*	Anemia (EI); diaphragmatic myositis (NI); endocarditis (NI); endomyocarditis - left ventricular; endocardial fibrosis complex (NI); eosinophilia (NI); fever (EI, NI); hyperglobulinemia (EI, NI); lethargy (EI, NI); lymphadenomegaly (EI); mild neurological signs (EI); pyogranulomatous myocarditis and uveitis, conjunctivitis, keratitis and corneal ulcers (NI); subclinical (EI, NI); thrombocytopenia (NI)	Endocarditis (NI); eosinophilia (NI); epistaxis(NI); fever (NI); granulomatous hepatitis (NI); granulomatous inflammation (NI); hyperglobulinemia (NI); hyperinsulinemic hypoglycemia syndrome (NI); ineffective erythropoiesis (NI); lymphadenomegaly (NI); monoclonal gammopathy (NI); peliosis hepatis (NI); subclinical (EI, NI); thrombocytopenia (NI); vasoproliferative lesions (NI)	Arthralgia; arthritis; bacillary angiomatosis; CSD; endocarditis; erythema; granulomatous hepatis; neuroretinitis; peliosis hepatis; pulmonary nodules; uveitis; vasoproliferative tumors
*B. vinsonii berkhoffii*	Endocardial fibrosis complex (NI); endomyocarditis - left ventricular; osteomyelitis (NI)	Anemia (NI); arrhythmias (NI); endocarditis (NI); epistaxis (NI); fever (NI); granulomatous lymphadenitis (NI); hemangiosarcoma (NI); myocarditis (NI); polyarthritis (NI); splenomegaly (NI); subclinical (EI, NI); thrombocytopenia (NI); uveitis (NI)	Endocarditis
*B. clarridgeiae*	NCR or subclinical	Endocarditis (NI); hepatic disease (NI)	CSD
*B. quintana*	NCR or subclinical	Endocarditis (NI); subclinical (NI)	Bacillary angiomatosis; endocarditis; fever; neuroretinitis; uveitis
*B. koehlerae*	Endomyocarditis - left ventricular; endocardial fibrosis complex	Endocarditis (NI); hyperinsulinemic hypoglycemia syndrome (NI); splenic disease (NI)	Endocarditis
*B. rochalimae*	NCR or subclinical (EI)	Endocarditis (NI); subclinical (EI)	Fever; splenomegaly
*B. washoensis*	NCR or subclinical	Endocarditis (NI)	Fever; myocarditis

^a^Details included in Table 6 are provided in [20–22, 29, 30, 39, 70, 91, 92, 96, 98, 99, 112, 127, 132, 136, 154, 160, 168–170, 289, 292–305]

^b^Pathology reported in dogs to date is mainly due to natural infection only

*Abbreviations*: *CSD* cat scratch disease, *EI* experimental infection, *NI* natural infection, *NCR* not clearly related (the reports did not completely prove the direct relation between the clinical findings and the *Bartonella* infection or the animals had subclinical infection)

Currently, dogs appear to be an accidental rather than reservoir host for *B. henselae*, which is supported by the fact that this is the most frequently documented *Bartonella* spp. detected in sick dogs [128]. *Bartonella henselae* DNA was also the predominant *Bartonella* spp. amplified and sequenced from dogs with splenic hemangiosarcomas [129]. To date, *B. henselae* is the only *Bartonella* spp. associated with peliosis hepatis in dogs and humans [130, 131]. *Bartonella henselae* has been associated with other disease manifestations in dogs (Table 6) such as pyogranulomatous lymphadenitis, hepatitis and pulmonary nodules, dermatitis, panniculitis and endocarditis [92, 93, 99, 132]. In humans, *B. henselae* causes cutaneous vasoproliferative lesions (bacillary angiomatosis) and parenchymal vasoproliferative lesions of the liver, spleen (bacillary peliosis), and less frequently other tissues, particularly in immunosuppressed individuals including transplant recipients, and HIV and cancer patients [14, 133] (Table 6).

*Bartonella vinsonii berkhoffii* was first isolated from a dog with endocarditis in 1993 [87]. In dogs, *B. vinsonii berkhoffii* infection has been associated with endocarditis, arrhythmias, myocarditis, granulomatous lymphadenitis and granulomatous rhinitis. Clinical cases of *B. vinsonii berkhoffii* infection in cats and humans have been rarely described in the literature and clinical findings are summarized in Table 6. Current studies indicate *Bartonella* spp. infections appear to be more pathogenic in dogs and humans than in cats, potentially reflecting differences in host evolutionary adaptations to these vector-borne organisms.

To date, few cases of canine bartonellosis have been reported from Europe (Fig. 2) or other continents when compared with the USA, and the clinical findings match those described in dogs from the USA. *Bartonella*-associated inflammatory cardiomyopathy was described in a dog from Italy [95]. *Bartonella* infection in association with panniculitis, polyarthritis and meningitis was reported in a dog from England [75]. In France, *B. henselae* was amplified from blood of a dog with fever of unknown origin and granulomatous lymphadenitis [134] as well as from saliva in a subclinical German dog owned by a human patient suffering angioedema due to *B. henselae* [135]. In Spain, *B. koehlerae* DNA was amplified from blood and mitral valve tissue of a dog with infective endocarditis [30] and *B. rochalimae*, *B. vinsonii berkhoffii* and *B. koehlerae* were detected by PCR in valve tissue or blood from eight out of 30 (26.6%) dogs with blood culture-negative endocarditis [136]. In another study seroreactivity to *B. henselae* was detected in a dog with a monoclonal gammopathy and *Bartonella* species DNA was amplified from splenic tissue [98].

## Diagnosis and identification methods

Accurate diagnosis of *Bartonella* infections remains challenging. Currently there is no diagnostic technique for which a negative result assures the absence of infection. The most frequently used techniques for the detection of acute and chronic infections are specialized microbiological culture techniques, polymerase chain reaction (PCR), immunohistochemistry (IHC) and serology [137].

Specialized culture techniques including lysis centrifugation, cell culture isolation and growth enrichment in insect biochemical composition growth media are the “gold standard” for confirmation of *Bartonella* infection. Optimal samples for microbiological culture include blood, cerebrospinal fluid [138], joint fluid [81], pathological effusions [138] and tissue biopsies [139]. In reservoir-adapted hosts such as rodents and cats and infrequently in accidental hosts (sick dogs or humans), *Bartonella* spp. can be cultured successfully with agar plates containing 5% defibrinated rabbit or sheep blood, that are maintained at 35 °C in a high humidity chamber with 5% CO_2_ concentration. Agar plate isolation requires prolonged incubation times: bacterial colonies may not be visible until 10–56 days after inoculation of the agar plate [140]. Because *Bartonella* spp. are fastidious, slow-growing bacteria, a negative blood culture or biopsy culture after a long incubation period does not exclude suspected *Bartonella* infection [141]. Furthermore, the patient can be intermittently bacteremic as documented in feline *B. henselae* experimental infections [59, 112]. Similarly, testing serial blood specimens collected over a 7-day period enhanced microbiological documentation of *Bartonella* as reported in humans [142]. BAPGM, an optimized insect cell medium, has been used in an enrichment culture platform to enhance the growth of *Bartonella* spp. prior to attempted subculture bacterial isolation. The BAPGM, prior to PCR testing, has been used to increase sensitivity for documentation of infection, thereby facilitating a diagnosis of bartonellosis in cats, dogs and humans [16]. The BAPGM platform combines enrichment culture of a clinical specimen in the liquid growth medium for a minimum of 7 days, followed by a highly sensitive PCR assay designed to amplify all known *Bartonella* spp. [142]. When testing cat blood samples, *B. henselae* and *B. clarridgeiae* can often be isolated effectively using agar plates; however, isolation of the same *Bartonella* spp. from sick cats, dogs, horses or human blood samples using an identical isolation approach lacks sensitivity. Although additional optimization of *Bartonella* spp. isolation is needed, the introduction of BAPGM has facilitated the successful isolation of *B. henselae* and several other *Bartonella* spp. from dog, horse, human and wildlife blood samples [142–145].

The most employed tissue for *Bartonella* detection by PCR is peripheral blood. However, PCR for *Bartonella* spp. detection and characterization can be also performed after DNA extraction from cerebrospinal fluid, joint fluid, bacterial cultures, oral swabs, lymph node or other tissue samples or aspirates depending on each individual clinical case. To avoid DNA denaturation by formalin fixation, it is advisable to store tissues for future testing or submit fresh or fresh frozen specimens for PCR amplification of *Bartonella* DNA. Once the PCR is positive for the genus *Bartonella*, the species can be determined using species-specific primers or optimally by DNA sequencing [146–149].

Seroconversion can be used to confirm acute *Bartonella* spp. infection by documenting a four-fold rise in antibody titer over a 2–3-week period [16]. To date, there has been minimal use of serology or other diagnostic modalities for testing cats or dogs with acute onset illness [56]. Serological tests used to detect antibodies include IFA, ELISA and western immunoblot [56]. Serological tests appear to have good specificity and can be used to confirm prior or ongoing infection, but due to poor sensitivity, serology is of more limited value for predicting bacteremia in dogs and potentially sick cats [69, 150]. In cats, high antibody titers often correlate with positive blood cultures or PCR amplification of *Bartonella* DNA directly from blood [140]. Alternatively, the inability to detect *B. henselae* antibodies appears to be predictive of the absence of bacteremia in healthy cats [151], but similar to dogs and humans, there are sick bacteremic cats that do not have detectable *Bartonella* spp. antibodies, for reasons that remain unclear [128]. It is important to note that only 50% of dogs infected with *B. vinsonii berkhoffii* and 25% of dogs infected with *B. henselae* have *Bartonella* specific IFA antibody reactivity to the respective organism. PCR amplification of organism-specific gene fragments is often diagnostically useful for *Bartonella* cases in which culture and serology results are negative [128].

Studies to date indicate that inflammatory lesions (e.g. pyogranulomatous inflammation) can be severe; however, few organisms are normally visualized [111]. Therefore, stains and techniques to better visualize bacteria in histological specimens are available such as Warthin-Starry staining or immunohistochemistry. *Bartonella* spp. as well as other bacteria such as *Helicobacter pylori* or *Legionella pneumophila* can be visualized in biopsied tissues using Warthin-Starry staining [152]. For this reason, other techniques like *Bartonella* immunohistochemistry, fluorescent *in situ* hybridization (FISH) and PCR can be used to confirm that the bacteria observed by Warthin-Starry staining of histopathological lesions are *Bartonella* spp. [153].

Immunohistochemistry, including confocal immunohistochemistry, has been used for the detection of *Bartonella* spp. in cat, dog and human tissues [38, 94, 153–157]. The principal advantage of immunohistochemistry over other antigen detection techniques is the ability to identify the organism directly in the tissue samples such as cardiac valves or lymphoid organs and thus more effectively establish correlations between antigen localization and histopathological lesions [158]. An immunoassay using two specific in-house *B. henselae* monoclonal antibodies (MAb) documented the intra-erythrocytic localization of this bacterium in three blood culture positive cats. That study concluded that direct fluorescence with a specific MAb is a sensitive, rapid and simple technique which could be useful for detecting *Bartonella* infections in healthy cats [159].

## Clinical decision making in light of diagnostic results

The definitive diagnosis of bartonellosis in cats, dogs and, based upon more recent literature, humans [62, 104] remains a clinical, microbiological and pathological challenge. Based on the broad spectrum of historical and clinical abnormalities, bartonellosis is often among differential diagnostic considerations for various clinical problems. However, in many clinical situations, bartonellosis is either not considered diagnostically or becomes a diagnosis after exclusion of other compatible disease entities. However, it is important for clinicians to attempt to achieve diagnostic confirmation prior to embarking upon a long duration antibiotic therapy. A positive therapeutic response to antibiotics, in conjunction with seroreactivity or positive culture or PCR results, provides indirect support for a definitive diagnosis of bartonellosis. Prior or ongoing administration of antibiotics and potentially immunosuppressive drugs can adversely affect serological and molecular diagnostic test results [56, 160]. According to the experience of the authors and current literature, *Bartonella* infection should be investigated using both serology, culture and/or molecular methods (PCR) in healthy pets when: (i) screening cats and dogs as blood donors [60]; (ii) in pets owned by inmunocompromised persons [161]; (iii) *Bartonella* infection has been diagnosed or is suspected in a pet owner [162]; and (iv) when there is a history of exposure to fleas, ticks, others arthopods or scratch or bite wound in sick pets [163]. Interpretation of various diagnostic results to guide clinical decision making are summarized in Table 7.

**Table 7:** Treatment decision based on culture, PCR and serology results in sick animals with suspected *Bartonella* infection [16, 59, 62, 104, 112, 128]

Diagnostic methods			*Bartonella* infection^a^	Treatment decisions options
Culture	PCR	Serology		
+	+	+	Confirmed	Treat
+	+	-	Confirmed	Treat
+	-	-	Confirmed	Treat
+	-	+	Confirmed	Treat
**-**	+	+	Confirmed	Treat
**-**	+	-	Confirmed	Treat
**-**	-	+	Bartonellosis not excluded; Repeat culture and PCR if the suspicion of clinical bartonellosis remains	Do not treat or treat empirically if disease progresses. Empirical treatment should not be routinely recommended
-	-	-	Bartonellosis not excluded; Repeat serology in 2–3 weeks or culture and PCR in a few days if the suspicion of clinical bartonellosis remains	Do not treat or treat empirically if disease progresses. Empirical treatment should not be routinely recommended

^a^Despite diagnostic confirmation of bartonellosis in cats and dogs, as listed in the table, vector-borne disease co-infections, co-morbidities and other differential diagnoses should be evaluated in conjunction with or prior to administration of antimicrobial drugs

*Key*: +, positive; -, negative

## Treatment

Antimicrobial therapy comprises the primary treatment modality and in most cases a combination of antibiotics is necessary to achieve disease resolution (Table 8). There is no standardized antibiotic protocol for treatment of bartonellosis in cats or dogs [164]. Data from controlled efficacy studies involving naturally-infected cats and dogs are lacking. While many antibiotics are effective *in vitro*, *in vivo* efficacy appears to vary among individual patients [25]. Treatments have varied depending upon the predominant tissue location of disease manifestations (e.g. endocard, brain, or blood stream infection).

**Table 8:** Reported treatments in cats and dogs

Host	Clinical *Bartonella* spp. manifestations/species	Treatment	Dose/duration	Reference^a^
Cats	Bacteremia and uveitis/*Bartonella* spp.	Doxycycline + Pradofloxacin	5 mg/kg PO q 12 h/4–6 weeks + 5 mg/kg PO q 12 h/4–6 weeks	[167]
		Doxycycline	10 mg/kg PO q 12–24 h/4–6 weeks	[170]
		Azithromycin	10 mg/kg PO q 24–48 h/ 7 days followed by every other day for 6–12 weeks	[169]
	Endocarditis/*B. henselae*	Marbofloxacin + Azithromycin	5 mg/kg PO q 24 h/6 weeks + 10 mg/kg PO q 24 h for 7 days and then q 48 h/6 weeks	[294]
	Osteomyelitis and polyarthritis/*B. vinsonii berkhoffii*	Amoxicillin-clavulanate + Azithromycin	62.5 mg PO q 12 h/2 months + 10 mg/kg PO q 48 h/3 months	[168]
Dogs	Splenic vasculitis, thrombosis and infarction/*B. henselae*	Doxycycline + Trimethoprim-sulfamethoxazole	5–10 mg/kg PO q 12 h/4 weeks + 23 mg/kg, PO q 12 h/6 weeks	[28]
	Neurological and ocular disorders/*Bartonella* spp.	Doxycycline + Enrofloxacin	5–15 mg/kg PO q 12 h + 5 mg/kg PO q 12 h/4–6 weeks	[169]
		Doxycycline + Rifampicin	5–10 mg/kg PO q 12 + 5 mg/kg PO q 24 h/ 4-6 weeks	
	Endocarditis/*B. koehlerae*	Ampicillin + Enrofloxacin	22 mg/kg PO q 8 h + 5 mg/kg PO q 12–24 h/4–6 weeks	[30]
	Hemangiopericytoma/*B. vinsonii berkhoffii*	Enrofloxacin	5 mg/kg PO q 12 h/4–6 weeks	[14]

^a^Details included in Table 8 are provided in references

*Abbreviations*: *q* every, *PO* oral administration

Most laboratory-based antibiotic treatment studies indicate that complete clearance of *Bartonella* spp. from cats has not been achieved with antibiotics studied to date (doxycycline, amoxicillin, amoxicillin-clavulanic acid, enrofloxacin, erythromycin and rifampicin) [59, 164–166]. Results of these studies were variable with bacteremia apparently being eliminated in some cats [167, 168]. Serum antibody titers typically decrease rapidly (3–6 months) and remain below the limits of detection in animals that have a positive treatment response, and have presumably eliminated the infection [2]. Treatment in sick cats is recommended when *Bartonella* spp. are confirmed diagnostically and compatible disease entities (e.g. endocarditis, encephalitis, myocarditis, fever and uveitis) are suspected or confirmed (Table 8). Because widespread use of antibiotics contributes to antimicrobial resistance among non-targeted bacteria, antibiotic treatment is not routinely recommended for healthy, *B. henselae* bacteremic cats, despite the risk of zoonotic transmission [167]. However, antibiotic treatment of bacteremic healthy cats living in a household with immunocompromised adults or young children is recommended. In these cases, treatment is aimed at decreasing bacterial load, minimizing the risk of additional vector exposure and thus decreasing the risk of transmission among pets or to humans.

An optimal protocol for treatment *Bartonella* spp. infection in dogs has also not been established. Use of an antibiotic capable of crossing lipid membranes and reaching high intracellular concentrations, such as amoxicillin, azithromycin, doxycycline and enrofloxacin is recommended [168–170]. Macrolides, like azithromycin, are effective but are not recommended as a first line antibiotic due to rapid development of resistance among *B. henselae* strains. Once genetically-mediated (mutation) resistance developed, *B. henselae* isolates were resistant to all macrolides [16]. For dogs with central nervous system involvement, a combination of doxycycline and rifampicin has been used successfully, but the use of rifampicin is not recommended in cats [167]. Aminoglycosides, used to treat human endocarditis, are recommended in conjunction with careful monitoring of renal function during the initial treatment of suspected *Bartonella* endocarditis or myocarditis in cats and dogs. A combination of doxycycline and amikacin represents a treatment option for *Bartonella* endocarditis in cats and dogs [16]. For dogs that are reasonably stable starting with one antibiotic (for example doxycycline at 5 mg/kg every 12 hours) and adding the second antibiotic 5–7 days later may help to avoid a potential Jarisch-Herxheimer-like reaction that appears to be related to rapid bacterial injury/death. The Jarisch-Herxheimer-like reaction is typically associated with lethargy, fever, occasionally vomiting and commonly occurs in cats and dogs at 4–7 days after starting antibiotics. If a Jarisch-Herxheimer-like reaction occurs, it is not recommended to interrupt or change antibiotics; supportive therapy and anti-inflammatory steroids for a few days may help dogs through this period [167].

General treatment recommendations for feline and canine bartonellosis based upon the literature and the authors’ experiences are summarized below:

- Diagnostic confirmation of clinical bartonellosis is recommended or a very high index of suspicion.
- Prolonged treatment periods (4–6 weeks) are recommended to avoid bacterial drug resistance and to achieve disease resolution.
- Antibiotics are currently the mainstay of treatment.
- It is not recommended to use macrolides as the first therapy option.
- Antibiotics combinations with various mechanisms of action, achieving therapeutic drug concentrations within cells and within plasma are needed to eradicate *Bartonella* infections.

## Preventative measures

As vaccines are not available to prevent infection, flea and tick control are the only successful measures to prevent this vector-borne infection in healthy animals [166], to decrease the dispersion of these bacteria among canine and feline populations, and to decrease the risk of zoonotic pathogen transmission to humans [65]. Cats and dogs should be protected from flea and tick infestations year-round by the regular use of acaricides in the form of collars, spot-on or spray-on or oral formulations [171]. Furthermore, both people and pets should avoid contact with stray dogs and cats. In the context of One Health, the authors support the future development of vaccines to protect pets against infection with *B. henselae* and *B. vinsonii berkhoffii* and thereby decrease reservoir potential and zoonotic risks.

In households with immunosuppressed persons or young children, if their pets are determined as bacteremic, antibiotic treatment and routine acaricide use are recommended for these pets. When acquiring a new cat or dog, into a household with immunocompromised individuals and children, choosing an adult animal will lower the possibility of acquiring a *Bartonella* spp. bacteremic pet [25, 65].

Blood transfusion has also been identified as a risk factor for the transmission of *Bartonella* infections. Screening of blood donors for *Bartonella* infections, should be considered [37, 60].

## Conclusions

Based upon the recent and ongoing discovery of novel *Bartonella* spp. in hosts such as bats [172, 173] and rodents [174], it is likely that additional *Bartonella* spp., in conjunction with their respective reservoir host and vector, will be described. Furthermore, as *Bartonella* spp. transmission routes are not fully understood, research efforts should focus on modes of transmission so that appropriate control measures can be implemented to prevent the pathogen transmission between animals and from animals to humans. *Bartonella* spp. seroprevalence rates in cats and dogs in Europe and other parts of the world do not correspond with the low number of reported clinical cases, especially in dogs, potentially because *Bartonella* infections are underdiagnosed. The limited number of reported cases of *Bartonella* spp. infection compromises our collective ability to establish a complete list of clinical conditions or specific pathologies related to this infection. In conclusion, more efforts are needed in both research and clinical settings to characterize the medical importance of *Bartonella* spp. infections in cats and dogs. Additionally, randomized case control studies are needed to assess treatment efficacy and to establish an optimal protocol for the treatment of chronic bartonellosis in cats, dogs and humans. Efforts to develop safe and effective vaccines are needed to protect pets and their families.

## Abbreviations

BAPGM: *Bartonella* alpha proteobacteria growth medium
CO_2_: Carbon dioxide
CSD: Cat scratch disease
EI: Experimental infection
ELISA: Enzyme-linked immunosorbent assay
FeLV: Feline leukemia virus
FISH: Fluorescent in situ hybridization
HIV: Human immunodeficiency virus
IFA: Indirect immunofluorescence assay
IgG: Immunoglobulin G
IHC: Immunohistochemistry
MAb: Monoclonal antibody
NA: Not applicable
NCR: Not clearly related
NI: Natural infection
NF: not found
nr: Not reported
PCR: Polymerase chain reaction
PO: Oral administration
q: Every
VEGF: Vascular endothelial growth factor
